# Prognostic value of quantitative fluorodeoxyglucose measurements in newly diagnosed metastatic breast cancer

**DOI:** 10.1002/cam4.119

**Published:** 2013-09-12

**Authors:** Gary A Ulaner, Anne Eaton, Patrick G Morris, Joshua Lilienstein, Komal Jhaveri, Sujata Patil, Maurizio Fazio, Steven Larson, Clifford A Hudis, Maxine S Jochelson

**Affiliations:** 1 Department of Radiology, Memorial Sloan-Kettering Cancer CenterNew York, New York; 2 Department of Radiology, Weill Cornell Medical CollegeNew York, New York; 3 Department of Epidemiology-Biostatistics, Memorial Sloan-Kettering Cancer CenterNew York, New York; 4 Breast Cancer Medicine Service, Memorial Sloan-Kettering Cancer CenterNew York, New York; 5 Department of Medicine, Weill Cornell Medical CollegeNew York, New York

**Keywords:** Breast cancer, FDG PET/CT, mean tumor volume, SUV _max_, total lesion glycolysis

## Abstract

The aim of this study was to determine the prognostic value of quantitative fluorodeoxyglucose (FDG) measurements (maximum standardized uptake value [SUV_max_], metabolic tumor volume [MTV], and total lesion glycolysis [TLG]) in patients with newly diagnosed metastatic breast cancer (MBC). An IRB-approved retrospective review was performed of patients who underwent FDG positron emission tomography (PET)/computed tomography (CT) from 1/02 to 12/08 within 60 days of diagnosis MBC. Patients with FDG-avid lesions without receiving chemotherapy in the prior 30 days were included. Target lesions in bone, lymph node (LN), liver, and lung were analyzed for SUV_max_, MTV, and TLG. Medical records were reviewed for patient characteristics and overall survival (OS). Cox regression was used to test associations between quantitative FDG measurements and OS. A total of 253 patients were identified with disease in bone (*n* = 150), LN (*n* *=* 162), liver (*n* = 48), and lung (*n* = 66) at the time of metastatic diagnosis. Higher SUV_max_ tertile was associated with worse OS in bone metastases (highest vs. lowest tertile hazard ratio [HR] = 3.1, *P* < 0.01), but not in LN, liver or lung (all *P* > 0.1). Higher MTV tertile was associated with worse OS in LN (HR = 2.4, *P* < 0.01) and liver (HR = 3.0, *P* = 0.02) metastases, but not in bone (*P* = 0.22) or lung (*P* = 0.14). Higher TLG tertile was associated with worse OS in bone (HR = 2.2, *P* = 0.02), LN (HR = 2.3, *P* < 0.01), and liver (HR = 4.9, *P* < 0.01) metastases, but not in lung (*P* = 0.19). We conclude measures of FDG avidity are prognostic biomarkers in newly diagnosed MBC. SUV_max_ and TLG were both predictors of survival in breast cancer patients with bone metastases. TLG may be a more informative biomarker of OS than SUV_max_ for patients with LN and liver metastases.

Measures of fluorodeoxyglucose (FDG) avidity are prognostic biomarkers in newly diagnosed metastatic breast cancer. Volumetric measurements, such as total lesion glycolysis (TLG), may be more informative biomarkers for survival than the more commonly used standardized uptake value (SUV).

## Introduction

Fluorodeoxyglucose positron emission tomography/computed tomography (FDG PET/CT) is an integral modality in the imaging of breast cancer. Preoperative FDG PET/CT staging of breast cancer patients with locally advanced disease alters patient management through detection of unsuspected nodal and distant metastases 1–4. Furthermore, the effectiveness of FDG PET/CT in monitoring breast cancer treatment response 5–9 and detecting recurrence 10–12 is well established. However, little is known about whether FDG avidity can be used as a prognostic tool in patients with newly diagnosed metastatic breast cancer (MBC) before therapy 13. We have recently published a series evaluating maximum standardized uptake value (SUV_max_) as a prognostic tool from data obtained in clinical reports 14. Limitations in prior studies include small cohort sizes, cohorts without histologic proof, and lack of consensus regarding the optimum method for quantifying FDG avidity.

Quantification of FDG avidity today is widely reported using SUV_max_, probably due to its high reproducibility with modern computer software. However, SUV_max_ has limitations, including its emphasis on only a single volumetric pixel (voxel) within a lesion, which makes it very susceptible to statistical noise 15. SUV_mean_ is much less susceptible to noise, but suffers from poor reproducibility depending on the number of included voxels within the region of interest (ROI) 15. Some have advocated a compromise between SUV_max_ and SUV_mean_, termed SUV_peak_, which measures a local average SUV value around the SUV_max_ 16. All three of these parameters may be limited by not incorporating the volume of metabolically active disease. To overcome this limitation, others have proposed measures such as metabolic tumor volume (MTV) and total lesion glycolysis (TLG) 17. TLG incorporates both intensity of FDG avidity as well as metabolic volume in its quantification 17. At our institution, SUV_max_ is most commonly reported; however, MTV and TLG are being investigated as independent biomarkers.

We hypothesized that FDG avidity would be a useful biomarker of overall survival (OS) in patients with newly diagnosed MBC. In this retrospective study, we examine quantitative measurements of FDG avidity (SUV_max_, MTV, and TLG) as predictors of OS in a large cohort of newly diagnosed MBC patients with a high percentage of histologically proven metastases.

## Material and Methods

This study was performed under Memorial Sloan-Kettering Cancer Center (MSKCC) Institutional Review Board approval. Inclusion criteria and methods for the retrospective review have been previously described 14. In brief, MSKCC databases were used to identify patients with FDG lesions on FDG PET/CT within 60 days of diagnosis of MBC. Patients receiving chemotherapy within 30 days prior to PET/CT were excluded. Medical records were used to collect data on standard prognostic variables and patient characteristics including estrogen receptor (ER)/progesterone receptor (PR)/human epidermal growth factor receptor 2 (HER2) expression, time from cancer diagnosis to metastasis, treatment, and OS defined as time from metastasis to date of death or last follow-up. PET/CT examinations were performed according to MSKCC clinical protocols and reviewed for FDG-avid reference lesions as previously described 14. PET/CT was performed on 4 GE and 1 Siemens hybrid PET/CT scanners, including acquisition of images from the mid skull to upper thigh ∼60 min after intravenous administration of 12–15 mCi of FDG. Patients fasted >6 h, and finger stick blood glucose levels were <200 mg/dL prior to injection. Spiral CT was obtained for attenuation correction at 60 mAs, 120–140 kVp, with a 5-mm slice thickness while the patient was free breathing. PET was acquired at 3–5 min per bed position using the 3D mode, typically six to seven bed positions.

**Figure 1:**
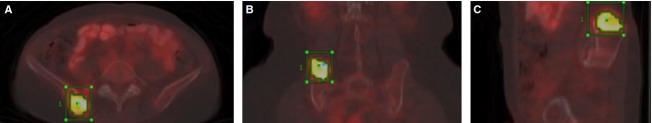
Measurement of SUV_max_, MTV, and TLG in reference lesions. Transaxial (A), coronal (B), and sagittal (C) hybrid FDG PET/CT images of a reference bone metastasis. A region of interest (ROI) was drawn for each reference lesion (green boxes) and checked for correct positioning in all three axial planes. The voxel with the greatest FDG avidity was marked (green dots) to confirm that this voxel was originating within the ROI. SUV_max_, MTV, and TLG were then obtained using GE AW Suite software. Biopsy demonstrated an osseous metastasis in this representative case. SUV_max_, maximum standardized uptake value; MTV, metabolic tumor volume; TLG, total lesion glycolysis; FDG PET/CT, fluorodeoxyglucose positron emission tomography/computed tomography.

Lesions were stratified by the most common sites of breast cancer metastases (bone, lymph node [LN], liver, and lung). Site-specific stratification was performed to account for technical factors, such as respiratory motion, which alter FDG-avidity measurements in individual organs uniquely 18. Local-regional nodes were only measured if the patient had another lesion that qualified as distant metastatic disease. All reference lesions were selected by a single investigator with 7 years PET/CT experience (G. A. U.). We previously described SUV_max_ by original report as a prognostic biomarker 14. In this study, PET/CT scans included in the prior analysis were reviewed and measurements of FDG avidity were obtained by G. A. U. and two assistants (J. L. and K. J.), who were all blinded to clinical data. First, SUV_max_ was recalculated and then measurements of MTV (cm^3^), and TLG (SUV_mean_ × cm^3^) were obtained from reference lesions by drawing regions of interest using GE AW Suite software (Fig. 1). Non-FDG-avid lesions were not measured, as lack of intravenous contrast, breathing motion artifacts, and reduced soft tissue resolution resulting from the limited CT performed as part of FDG PET/CT reduce the sensitivity of detecting non-FDG-avid CT lesions as compared to standard CT examinations.

SUV_max_ was the single voxel within the ROI with the greatest SUV. MTV was defined as the cubic centimeter volume of voxels with SUV >42% of SUV_max_, as described in a prior publication 17. TLG was defined as the product of MTV and the SUV_mean_ of voxels within the MTV 17. All three measurements were recorded as maximums for each metastatic site. In some instances, more than one lesion per site may have been used to record values for SUV_max_, MTV, and TLG.

### Statistics

OS was calculated from the date of MBC to death or last date of follow-up and was analyzed using the Kaplan–Meier method or Cox proportional hazards models. Analyses investigating the association between FDG avidity and OS were stratified by the four metastatic sites (bone, LN, liver, and lung). Patients with lesions at multiple metastatic sites were included in analyses for each site. Tertiles were defined at each site for SUV_max_, MTV, and TLG (Table 1). Modeling these parameters as tertiles allowed flexibility in the relationships that could be detected while keeping the risk of overfitting relatively low (lower than quartiles, quintiles, etc.). If univariate analyses of tertiles of SUV, MTV or TLG were significant, then multivariate models adjusting for known prognostic variables were constructed. The multivariate analyses were controlled for ER/PR/HER2 status, visceral metastases, grade, and histology. It was not possible to fit multivariate models for patients with liver metastases due to the small number of events. As a sensitivity analysis, a single model incorporating known prognostic variables and present/absent variables for each disease site was built (base model). The likelihood ratio test was then used to assess the significance of adding each of the following groups of four covariates to the base model in three separate analyses: (1) interactions between each site and site-specific SUV, (2) interactions between each site and site-specific MTV, and (3) interactions between each site and site-specific TLG. For these models all PET parameters were log-transformed to enhance normality. All statistical analyses were performed with SAS 9.2 (SAS Institute Inc., Cary, NC) and R 2.11.1 (The R Foundation for Statistical Computing, Vienna, Austria) statistical software. A *P*-value below 0.05 was considered statistically significant.

**Table:** Cut-offs used to define site-specific tertiles

	First tertile	Second tertile
Bone		
SUV (gram/mL)	6.0	9.7
MTV (cm^3^)	4.7	10.2
TLG (gram)	18.8	54.7
LN		
SUV	6.3	10.4
MTV	3.6	7.9
TLG	14.6	41.2
Liver		
SUV	6.2	12.8
MTV	7.9	15.3
TLG	32.3	100.6
Lung		
SUV	4.1	8.1
MTV	2.3	5.2
TLG	5.2	19.3

SUV, standardized uptake value; MTV, metabolic tumor volume; TLG, total lesion glycolysis; LN, lymph node.

## Results

As previously described, we identified 285 patients who underwent PET/CT within 60 days of diagnosis of MBC, with at least one FDG-avid lesion on PET/CT (Fig. 1). Following review of PET/CT scans, 32 cases were excluded (11 patients had PET only rather than PET/CT and 21 patients did not have measurable SUV), leaving a cohort of 253 patients. Overall, 228 of 253 (90%) of the patients had pathology confirming the diagnosis of metastatic disease. The median age range of patients was 57 (range 27–90). Sites of disease on FDG PET/CT at the time of metastatic diagnosis were LN (*n* = 162), bone (*n* = 150), liver (*n* = 48), and lung (*n* = 66), with 129 patients presenting with more than one organ site of metastasis. Median follow-up among survivors was 40 months (range 0.2–102.2 months) and 152 (60%) patients died. The median OS was 40 months. The number of deaths for patients with disease at each site was as follows: 108 LN, 87 bone, 39 liver, 39 lung.

### Clinical and histologic features and OS

Currently utilized histologic prognostic indicators for breast cancer include ER, PR, and HER2 expression, as well as histologic subtype (ductal, lobular) and tumor grade. The presence of visceral metastases (lung, liver) and shorter time from initial diagnosis to metastases are also associated with an adverse outcome in breast cancer 19. The hazard ratios for OS for these histologic and clinical features in the patient cohort are shown in Table 2, and are similar to the prior report 14. As expected, there was a statistically significant correlation between ER/PR/HER2 expression and OS, with triple-negative (ER-, PR-, and HER2-negative) tumors having the worst prognosis (*P* < 0.01). Time period of less than 5 years from primary diagnosis to metastasis was also associated with worse survival (*P* < 0.01). Neither histologic subtype nor tumor grade was significantly associated with OS. The presence of visceral metastases was correlated with poor OS (*P* = 0.03). Patients treated with targeted therapy (including with endocrine therapy or chemotherapy) or chemotherapy alone in the first-line setting had significantly decreased survival (*P* < 0.001) compared to patients treated with endocrine therapy.

**Table:** Clinical and histologic characteristics as prognostic variables in patients with newly diagnosed metastatic breast cancer (*N* = 253)

Variable	*N* (%)	*P*-value	HR (95% CI)
ER/PR and HER2 expression	246	<0.001^1^	
ER or PR+, HER2−	142 (58%)		Reference
HER2+	54 (22%)		1.11 (0.73–1.69)
Triple-negative	50 (20%)		3.04 (2.07–4.44)
Grade	219	0.11	
Grade 1 or 2	44 (20%)		Reference
Grade 3	175 (80%)		1.45 (0.92–2.30)
Histology	248	0.76	
Ductal	214 (86%)		Reference
Lobular	17 (7%)		1.20 (0.66–2.17)
Other	17 (7%)		0.89 (0.48–1.65)
Visceral metastases (lung and/or liver)	253	0.03^1^	
Absent	150 (59%)		Reference
Present	103 (41%)		1.41 (1.03–1.95)
Time from primary diagnosis to metastases	253	<0.001^1^	
More than 5 years	71 (28%)		Reference
3–5 years	37 (15%)		1.70 (1.00–2.89)
3 months–3 years	80 (32%)		2.44 (1.58–3.76)
Metastatic at diagnosis (<3 months)	65 (26%)		1.56 (0.97–2.49)

CI, confidence interval; HR, hazard ratio; *N*, number of patients evaluated; ER, estrogen receptor; PR, progesterone receptor; HER2, human epidermal growth factor receptor 2.

Indicates statistically significant values.

### Univariate analysis of FDG-avidity measurements and OS

We hypothesized that quantitative measurements of FDG avidity would correlate with OS in newly diagnosed MBC. The hazard ratios for OS for quantitative measurements of FDG avidity by metastatic site are shown in Table 3. We examined the concordance in classification between the tertiles of each marker and found that there was substantial heterogeneity in each marker within a fixed level of a different marker (results not shown), indicating that different markers may hold different information.

**Table:** SUV_max_, MTV, and TLG as prognostic variables in bone, LN, liver, and lung metastases

Variable	Bone (*N* = 150)			Lymph node (*N* = 162)				
	*P*-value	HR	*P*-value	HR
SUV_max_	<0.01^1^		0.14	
Low tertile		Reference		Reference
Middle tertile		2.29 (1.29–4.06)		1.59 (0.98–2.55)
High tertile		3.11 (1.80–5.39)		1.47 (0.91–2.39)
MTV	0.22		<0.01^1^	
Low tertile		Reference		Reference
Middle tertile		1.60 (0.93–2.75)		1.67 (1.00–2.79)
High tertile		1.50 (0.85–2.62)		2.38 (1.46–3.88)
TLG	0.02^1^		<0.01^1^	
Low tertile		Reference		Reference
Middle tertile		1.44 (0.84–2.47)		1.69 (1.02–2.81)
High tertile	–	2.15 (1.26–3.67)	–	2.28 (1.39–3.73)
	Liver (*N* = 48)			Lung (*N* = 66)				
*P*-value	HR	*P*-value	HR
SUV_max_	0.14		0.15	
Low tertile	–	Reference	–	Reference
Middle tertile	–	2.10 (0.96–4.60)	–	1.25 (0.56–2.80)
High tertile	–	1.91 (0.84–4.31)	–	2.11 (0.96–4.62)
MTV	0.02^1^		0.14	
Low tertile	–	Reference	–	Reference
Middle tertile	–	1.35 (0.59–3.06)	–	1.20 (0.52–2.79)
High tertile	–	2.95 (1.32–6.58)	–	2.06 (0.94–4.53)
TLG	<0.01^1^		0.19	
Low tertile	–	Reference	–	Reference
Middle tertile	–	2.14 (0.93–4.92)	–	1.11 (0.49–2.53)
High tertile	–	4.87 (2.00–11.89)	–	1.91 (0.88–4.13)

Hazard ratios (HR) are reported as estimate (95% confidence interval). SUV_max_, maximum standardized uptake value; MTV, metabolic tumor volume; TLG, total lesion glycolysis; LN, lymph node.

Indicates statistically significant values.

### Maximum standardized uptake value

A statistically significant correlation was seen between SUV_max_ tertile and OS in bone metastases (*P* < 0.01), with greater SUV_max_ values resulting in a greater risk of death. Statistical significance of SUV_max_ tertile was not reached in the other metastatic sites, LN (*P* = 0.14), liver (*P* = 0.14), and lung (*P* = 0.15).

### Metabolic tumor volume

A statistically significant association was seen between MTV tertile and OS in LN (*P* < 0.01) and liver (*P* = 0.02) metastases; with a higher tertile conferring a higher risk of death (Table 3). Statistical significance of MTV tertile was not reached in bone (*P* = 0.22) or lung (*P* = 0.14).

### Total lesion glycolysis

**Figure 2:**
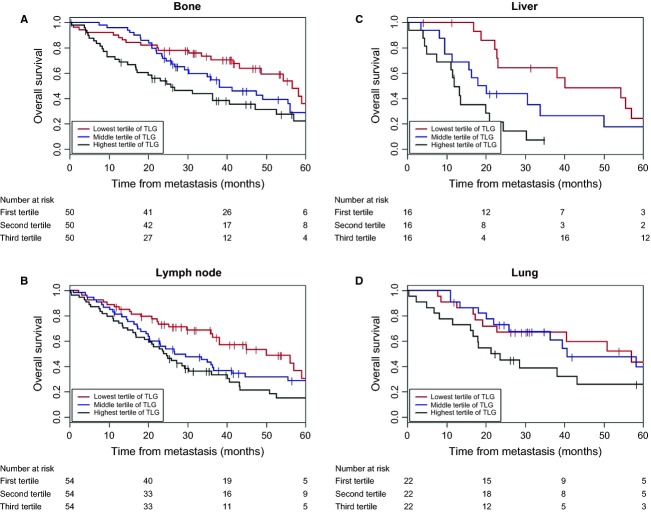
Kaplan–Meier curves of overall survival (OS) according to total lesion glycolysis tertiles (TLG) in (A) bone, (B) lymph node, (C) liver, and (D) lung. Time point zero is defined as the date of diagnosis of metastatic disease. Number of patients at risk for death at selected time points is displayed below each graph.

A statistically significant correlation was seen between TLG tertile and OS in bone (*P* = 0.01), LN (*P* < 0.01), and liver (*P* < 0.01) metastases (Table 3). Statistical significance of TLG tertile was not reached in lung (*P* = 0.20). A visual representation of this relationship can be seen in Figure 2 where the highest tertile of TLG experienced the shortest survival for each metastatic site.

### Multivariate analysis of FDG-avidity measurements and OS

Multivariate analyses were performed on the statistically significant univariate analyses (SUV_max_ in bone metastases, MTV in LN metastases, TLG in bone metastases, and TLG in LN metastases). Multivariate analyses were not performed for MTV in liver metastases or TLG in liver metastases due to insufficient sample sizes (*n* = 48 for liver metastases). The multivariate analyses were controlled for ER/PR/HER2 status, visceral metastases, grade and histology. Time from primary diagnosis was not included as a covariate in multivariate models because it was strongly related to ER/PR/HER2 status.

### SUV_max_ of bone metastases

In a multivariate model, SUV_max_ tertile (*P* < 0.01), ER/PR/HER2 (*P* < 0.01) and presence of visceral mets (*P* = 0.05) were significantly associated with OS in patients with bone metastases. Patients with values in the middle and high tertiles of SUV, respectively, had 2.28 (95% CI: 1.2, 4.32) and 3.13 (95% CI: 1.62, 6.03) times the hazard of death compared to patients with values in the lowest tertile. Grade (*P* = 0.10) and histology (*P* = 0.66) were not independent predictors of OS.

### MTV of LN metastases

Tertiles of MTV were not associated (*P* = 0.28) with OS in a multivariate model of patients with LN metastases after adjusting for subtype, grade, histology, and presence of visceral metastases. Only ER/PR/HER2 status was significantly associated with death in this model; patients with triple-negative disease experienced 2.27 (95% CI: 1.33, 3.88) times the hazard of death compared to patients with HER2-negative, ER- and/or PR-positive cancer.

### TLG of bone and LN metastases

Multivariate results for TLG in patients with bone metastases are given in Table 4. After adjusting for standard prognostic variables, TLG tertile remained significantly associated with OS (*P* = 0.02) in patients with bone metastases, with the highest tertile of TLG conferring a 2.19-fold (95% CI: 1.17–4.07) increase in the hazard of death compared to the lowest tertile. Of the standard prognostic variables, ER/PR/HER2 and presence of visceral mets were significantly associated with OS. Additionally, a multivariate model was built to investigate the association between TLG and selected variables and OS in patients with LN metastases. TLG tertile was marginally significant (*P* = 0.09) and values in the middle tertile conferred 1.62 times (95% CI: 0.9, 2.9) the hazard of death while values in the highest tertile conferred 1.92 times (95% CI: 1.06, 3.46) the hazard of death, compared to values in the lowest tertile. ER/PR/HER2 was strongly associated with OS (*P* < 0.01), with patients with triple-negative breast cancer experiencing a 2.21-fold (95% CI: 1.28, 3.81) increase in the hazard of death compared to those with HER2-negative, ER- and/or PR-positive cancer. Grade (*P* = 0.713), Histology (*P* = 0.583) and presence of visceral metastases (*P* = 0.359) were not significantly associated with survival.

**Table:** Multivariate analysis of TLG and overall survival in bone metastases

Variable	*P*-value	Hazard ratio
TLG	0.02^1^	
Lower tertile		Reference
Middle tertile		1.09 (0.58–2.04)
Upper tertile		2.19 (1.17–4.07)
ER/PR and HER2	<0.01^1^	
ER or PR+, HER2−		Reference
HER2+		0.89 (0.47–1.70)
Triple-negative		2.65 (1.43–4.92)
Grade (Grade 2 vs. 3)	0.08	1.73 (0.93–3.22)
Histology	0.44	
Ductal		Reference
Lobular		0.57 (0.17–1.88)
Other		1.43 (0.59–3.46)
Visceral metastases (+ vs. −)	<0.01^1^	2.14 (1.27–3.58)

Hazard ratios (HR) are reported as estimate (95% confidence interval). TLG, total lesion glycolysis; ER, estrogen receptor; PR, progesterone receptor; HER2, human epidermal growth factor receptor 2.

Indicates statistically significant values.

### Sensitivity analysis

We fit a model that contained standard prognostic variables and binary variables indicating the location of the metastasis. Using this as a base model, we found that SUV measurements (incorporated in the model as an interaction between the natural log of SUV and location of metastasis) were significantly associated with OS (*P* = 0.029), as was the addition of MTV (*P* < 0.0001) and TLG (*P* < 0.0001). For every metastasis site/PET parameter combination, the hazard ratio associated with the PET parameter was greater than one, although not all associations were significant. In the SUV model, only SUV from bone was significantly associated with survival (*P* = 0.0236, p for lung liver and LN all >0.15). In the MTV model, an association was seen in liver, bone and LN (all *P* < 0.015) but not lung (*P* = 0.098). TLG from liver, bone, and LN lesions were strongly associated with survival (all *P* < 0.002) but TLG from lung was not (*P* = 0.0152). These results are similar to those found in the metastatic site-specific models presented in the sections above.

## Discussion

The rationale for using FDG avidity as a possible prognostic marker derives from prior studies demonstrating FDG avidity correlates with known histopathological and immunohistochemical markers of aggressive breast cancer biology, including ER/PR/HER2 status, histologic subtype, and tumor grade 20–23. Thus, higher FDG avidity may be a marker for a more aggressive disease.

Unlike SUV_max_ which relies on a single voxel of information, TLG provides information on both volume of disease and intensity of FDG avidity. Thus, while SUV_max_ measurements are easy to report, there may be indications where more comprehensive measurements of FDG avidity, such as TLG, will be more valuable. Recent reports have demonstrated the prognostic value of TLG in oral cavity, oropharyngeal squamous cell cancer, and non-small cell lung cancer 24–26.

In our large retrospective study, we evaluated the prognostic value of FDG avidity in newly diagnosed MBC, stratified by metastatic site. In univariate analyses, TLG was associated with OS in the greatest number of sites. Statistically significant associations were found between OS and SUV_max_ in bone metastases, MTV in LN and liver metastases, and TLG in bone, LN, and liver metastases. In site-specific multivariate analyses, SUV_max_ and TLG were independent predictors of OS in bone metastases, and TLG was marginally significant in LN metastases. In a multivariate model combining data from all sites, liver and LN TLG were both significantly associated with survival; however, liver and LN SUV were not.

The primary analyses in this study stratified metastatic disease by organ of involvement (bone, LN, liver, and lung). This was performed to account for technical factors that often affect measurements of FDG avidity, the most significant being respiratory motion. FDG PET scans performed with free breathing result in imprecise measurements of FDG avidity, as the lesion moves due to respiratory motion during acquisition of images 18. This may have pronounced effects on measurements for lung lesions. Indeed, in this study measurements incorporating intensity of FDG avidity (SUV_max_, TLG) were lower in the lungs than for other metastatic sites (Table 1). We hypothesize that respiratory motion resulted in lower quantitative FDG measurements in lung metastases.

Lung metastases were the only metastatic site where there was not a statistically significant association between TLG and OS. As discussed above, the problem of respiratory motion during acquisition of images limits the accuracy of measurements for lung lesions. We hypothesize that respiratory motion resulted in inferior measurements of FDG avidity, thus producing inferior input values for statistical analyses.

The strengths of this study include the large cohort size, high percentage of biopsy proven metastatic disease, long term of clinical follow-up, and uniform collection of data from the original FDG PET/CT examinations. The extensive databases at MSKCC allowed for identification of over 250 patients for our cohort, much larger than prior studies. More than 90% of the patients in our cohort had biopsy proof of MBC, which is often not addressed in prior studies. As the median survival of MBC is less than 3 years 27, our median follow-up of 40 months provides extensive data. Careful data collection from the original PET/CT exams minimized misclassification of lesions and improved selection of lesions with the maximum FDG measurements of interest.

The weaknesses of the study include the retrospective study design, possible selection bias, non-uniform treatment regimens, lack of histologic proof for all selected reference lesions, non-validated cutoffs for PET parameters and the large number of variables tested. Retrospective studies introduce inherent biases which are difficult to overcome. There may have been selection bias in the cohort, as not all patients with MBC underwent PET/CT during the defined time period. Patients in the cohort received different treatment regimens, which may have affected survival. Although more than 90% of patients had biopsy proof of MBC, it cannot be assumed that all the reference lesions selected represented metastatic sites. Our cutoffs were chosen based on tertiles in our data and have not been validated or used in another study. Finally, many models were fit and no procedure was used to strictly control type I error; results presented here are meant to be exploratory and hypothesis-generating ones.

In conclusion, in patients with newly diagnosed MBC, measures of FDG avidity (SUV_max_, MTV, and TLG) are statistically significant prognostic variables. These results build on results from our previous report, in which SUV_max_ by original radiology report was associated with OS 14. This study suggests that TLG may be a more informative biomarker than the more commonly reported SUV_max_ for patients with LN and liver metastases. TLG was significant for OS in LN metastases in univariate models and marginally significant in multivariate models. TLG was significant for OS in liver metastases in univariate analyses, but multivariate analyses were not performed due to insufficient numbers.

## Conflict of Interest

None declared.
